# Riechstörungen evidenzbasiert diagnostizieren und behandeln. Der unterschätzte Sinn – neue Erkenntnisse belegen die Bedeutung und Leistungsfähigkeit des menschlichen Geruchssinns.

**DOI:** 10.1007/s10354-021-00895-x

**Published:** 2021-12-01

**Authors:** Christian A. Müller, Bertold Renner

**Affiliations:** 1 Klinik für Hals‑, Nasen- und Ohrenkrankheiten, Kopf- und Halschirurgie, Medizinische Universität Wien, Spitalgasse 23, 1090 Wien, Österreich; 2 Institut für Experimentelle und Klinische Pharmakologie und Toxikologie, Friedrich-Alexander-Universität Erlangen-Nürnberg, 91054 Erlangen, Deutschland; 3 Institut für Klinische Pharmakologie, Medizinische Fakultät Carl Gustav Carus, Technische Universität Dresden, Fetscherstraße 74, 01307 Dresden, Deutschland

**Keywords:** Anosmie, Hyposmie, Parosmie, Riechverlust, Riechtraining, Anosmia, Hyposmia, Parosmia, Smell loss, Smell training

## Abstract

Zunehmende Evidenz belegt, dass der menschliche Geruchssinn eine große Bedeutung für die Gesundheit und Lebensqualität einnimmt sowie eine besondere Leistungsfähigkeit aufweist. Durch die häufig auftretende Riechstörung im Rahmen der Infektion mit SARS-CoV‑2 rückt das klinische Interesse in der Behandlung von Patienten mit Verlust des Geruchs- und Geschmackssinnes in den Vordergrund. Der vorliegende Artikel beleuchtet wichtige Aspekte in der Diagnose und Therapie der chemischen Sinne.

## Abstract (en)

Recent research shows that the human sense of smell seems to be more efficient than previously believed and to have a major impact on our health condition and quality of life. During the COVID-19 pandemic, an increased clinical interest has become evident for the SARS-CoV-2 related impact on olfactory function. This article highlights important aspects in the diagnosis and therapy of the chemical senses.

## Aktuelle Aspekte zu COVID-19

Nach Beginn der Corona-Pandemie Ende 2019 in China und danach im Rest der Welt, trafen mit nur wenigen Wochen Verzögerung die ersten Berichte über das Auftreten von Riechstörungen im Rahmen der Infektion mit SARS-CoV‑2 ein. Ein deutlicher Anteil (ca. 30–65 %) der symptomatischen Patienten berichteten über eine plötzlich einsetzende Anosmie, die neben den typischen Symptomen der COVID-19-Erkrankung, wie Fieber, Husten, Atemnot, Halsschmerzen, auch als alleiniges Symptom auftrat [1]. Folglich wurde der Verlust des Riechvermögens im Frühjahr 2020 von den medizinischen Fachgesellschaften und Behörden als hinweisendes Warnsignal auf das Vorliegen einer COVID-19-Erkrankung definiert [2]. Die Dauer der Riechstörung wurde anfangs als reversibel und kurz beschrieben, bald wurde jedoch klar, dass ein Teil der betroffenen Patienten auch noch nach längerer Zeit an dem Verlust des Geruchssinns leiden. So konnte auch unsere Forschungsgruppe bereits im Frühjahr 2020 über hundert Betroffene mit persistierendem Riechverlust in eine Langzeit-Beobachtungsstudie einschließen. Erst in den nächsten Jahren ist zu erwarten, die Langzeitprognose aufzuklären.

Die demographischen Daten unserer Patientengruppe mit Riechverlust nach Infektion mit SARS-CoV‑2 zeigen in Übereinstimmung mit Daten andere Arbeitsgruppen, dass bei diesen Patienten mit ambulantem, leichteren Krankheitsverlauf, ein jüngeres Alter und das weibliche Geschlecht überwiegen, wohingegen Patienten mit der Notwendigkeit einer stationären Aufnahme vermehrt ein höheres Alter und männliches Geschlecht mit weniger häufigem Riechverlust aufweisen [3]. Dies legt die wissenschaftliche Fragestellung nahe, ob das „Riechsystem“ einen entscheidenden Prognose- oder Einflussfaktor im Rahmen der Infektion mit SARS-CoV‑2 (oder auch mit anderen Krankheitserregern) hat, zumal die Abnahme des Riechvermögens mit höherem Alter (Presbyosmie) belegt ist und Frauen im Schnitt Männern in ihrer Riechleistung überlegen sind [4]. Im Tierversuch konnte bereits eine sog. „ultraschnelle Immunantwort“, die durch Viren ausgelöst wird, nachgewiesen werden [5]. Dabei führt die Infektion der Riechschleimhaut mit bestimmten Viren zu einer systemischen immunologischen Abwehr.

Ob eine über die Riechschleimhaut nach Viruskontakt getriggerte Immunantwort beim Menschen ebenfalls erfolgt, und damit eventuell ein besseres Riechvermögen eine bessere Widerstandsfähigkeit gegenüber einer Vielzahl verschiedener Erkrankungen bedeutet, ist noch unklar. Erwiesen ist jedoch seit längerer Zeit, dass Krankheitserreger über die Riechnervenzellen ins Gehirn eindringen können, da die Riechnerven eine direkte Verbindung der Außenwelt mit dem Gehirn darstellen [6]. Somit schützen die nach Infektion mit Viren abgestorbenen Riechnervenzellen das Gehirn vor einer potenziell gefährlichen Infektion. Dies geschieht somit in der Regel auf Kosten des Riechvermögens, das im günstigen Fall nur passager vermindert ist (Hyposmie), bei Verlust der sich regenerierenden Reservezellen jedoch auch dauerhaft verloren sein kann (Anosmie). Die betroffenen Patienten erleiden dadurch meist eine deutliche Einschränkung der Lebensqualität [7]. Außerdem ist durch Studien belegt, dass Patienten mit Hyposmie und Anosmie signifikant häufiger durch die verminderte Wahrnehmung von Feuer/Rauch, austretendem Gas oder verdorbenen Lebensmittel gefährdet sind [8].

Es gibt außerdem aus einer aktuellen Studie zumindest einen indirekten Hinweis auf die Bedeutung des Riechvermögens für die Gesundheit des Menschen, da ein besseres Riechvermögen in Zusammenhang mit einem längeren Überleben gefunden wurde [9]. Ob umgekehrt ein verminderter Geruchssinn an sich als prognostisch ungünstig aufzufassen ist, oder lediglich als Folge bestehender oder durchgemachter Erkrankungen aufzufassen ist, müssen weitere Studien zeigen.

## Das olfaktorische System im Fokus der Wissenschaft

Nicht erst seit COVID-19 rückte der Geruchssinn in das weltweite Forschungsinteresse. So wurden olfaktologische Forschungsarbeiten 2004 mit dem Nobelpreis für Linda Buck und Richard Axel prämiert [10]. Es wurde die Arbeit von 1991 ausgezeichnet, in der die beiden Wissenschaftler die Riechrezeptoren kodierende Genfamilie beschrieb, die beim Menschen mehr als 1 % des gesamten Genoms ausmacht (ca. 350 Riechrezeptoren) [11]. Durch die Erregung mehrerer Riechrezeptoren durch einen Duftstoff und die Tatsache, dass ein olfaktorisches Rezeptorneuron lediglich einen spezifischen Rezeptor exprimiert, entstehen bei der Wahrnehmung von Duftstoffen jeweils einzigartige „Duftlandkarten“. Theoretische Berechnungen auf Basis experimenteller Erkennungsraten von Duftstoffgemischen durch Probanden, haben ergeben, dass der Mensch in der Lage wäre, mehr als tausend Milliarden Duftstoffe zu unterscheiden [12].

Die erstaunlichen Fähigkeiten des Menschen bei der Wahrnehmung von Gerüchen, wurde in einer kürzlich erschienenen Übersichtsarbeit verdeutlicht (so sind wir bei einigen Duftstoffen deutlich empfindlicher als sog. Makrosmatiker wie z. B. Mäuse, deren Überleben von einem intakten Geruchssinn abhängt) [13]. McGann beschreibt sehr anschaulich, wie der Mythos eines unterentwickelten Geruchssinns beim Menschen in der Wissenschaft und somit auch im Bewusstsein der Bevölkerung verankert wurde. So fußt diese fehlerhafte Zuschreibung beim Menschen auf den philosophischen und theoretischen Arbeiten Paul Brocas und Sigmund Freuds, der insbesondere die „höherstehende“ Entwicklung des Menschen den „niederen“ Instinkten der Tierwelt gegenübergestellt und somit die Rückbildung des Geruchssinns des Menschen ohne entsprechende Untersuchungen angenommen hatte. Spätere Forscher haben diese Ansichten ohne Hinterfragung und v. a. ohne die Durchführung von Experimenten übernommen, womit erst in neuerer Zeit die entsprechende wissenschaftliche Aufarbeitung in anerkannten Zeitschriften erfolgte. So hat Anfang der 2000er Jahre eine israelisch-amerikanische Forschergruppe experimentell die Fähigkeit des Menschen zum sog. scent-tracking (Folgen einer Duftspur ohne Zuhilfenahme andere Sinne außer dem Geruchssinn) belegt [14].

Eine besonders wichtige Funktion hat der menschliche Geruchssinn bei der Wahrnehmung von Speisen und Getränken, da erst der retronasale Duftstofftransport von der Mundhöhle über den Nasenrachen zur Riechschleimhaut im Nasendach den Feingeschmack vermittelt. Wir schmecken daher v. a. mit der Nase, da über die Zunge nur die Geschmacksqualitäten süß, sauer, salzig, bitter und umami (Glutamat) wahrgenommen werden. Die weiteren Sinne, die an der Wahrnehmung des Feingeschmacks (engl. flavor) beteiligt sind, sind die trigeminale Wahrnehmung (Temperatur, Schärfe, Konsistenz) sowie das Auge (wir essen auch mit den Augen) und das Gehör (z. B. das Knacken von Chips). Also sind bei der Wahrnehmung des Essens alle fünf Sinne beteiligt [15].

## Die Anamnese bei Riechstörungen

Patienten mit subjektiver „Schmeckstörung“ müssen im Rahmen der spezifischen Anamnese nach den unterschiedlichen Sinneskanälen befragt werden. Obwohl einer „Schmeckstörung“ oft ein verminderter Geruchssinn zugrunde liegt, schließt umgekehrt ein vom Patienten berichteter unbeeinträchtigter Feingeschmackssinn (z. B. die Wahrnehmung von Gewürzen etc.) eine Riechstörung nicht aus. Eigene Untersuchungen haben ergeben, dass bis zu 12 % der Patienten mit Anosmie keine Beeinträchtigungen beim Essen angeben. Bei diesen Patienten scheinen die anderen Sinneskanäle die fehlenden Anteile des Geruchssinns an der Flavor-Wahrnehmung zu ersetzen sowie unbewusste Erinnerungsvorgänge beteiligt zu sein [16].

Ein zentraler Anteil der Anamnese und der nachfolgenden Untersuchungen betrifft die HNO-spezifische Diagnostik möglicher sinunasaler Erkrankungen. Hierzu gehören an erster Stelle die allergische Rhinitis (mit den Symptomen der verstopften und rinnenden Nase, Niesen, Nasen- und Augenjucken) sowie die chronische Sinusitis mit oder ohne Polyposis nasi (mit den Symptomen der verstopften Nase, Schleimsekretion auch post-nasal, Druckgefühl oder Schmerzen im Bereich der Nasennebenhöhlen, Riechverlust).

Im Rahmen der Anamnese sollte immer nach der spezifischen Wahrnehmung der Grundgeschmacksqualitäten gefragt werden („nehmen Sie Zucker, Zitrone, etc. auf der Zunge wahr?“). Untersuchungen haben ergeben, dass die subjektiv unveränderte Wahrnehmung der Grundgeschmacksqualitäten eine echte Schmeckstörung weniger wahrscheinlich macht [17]. Eine Schmecktestung sollte dann erfolgen, wenn der Patient eine Beeinträchtigung in der Wahrnehmung der Grundgeschmacksqualitäten angibt. Dazu eignen sich validierte Testverfahren, wie die in Erlangen entwickelten Schmeckstreifen („Taste Strips“), die pro Qualität in vier verschiedenen Konzentrationen angeboten werden, womit eine Quantifizierung des Schmeckvermögens ermöglicht wird [18]. Alternativ können entsprechende Schmecklösungen hergestellt werden.

Bei den echten Störungen des Geschmackssinns, sind eine Reihe an Differentialdiagnosen zu beachten. Typischerweise können Medikamente zur Veränderung der Wahrnehmung von süß, sauer, salzig, bitter führen. Die postulierten Mechanismen sind vielfältig. So können Veränderungen der Speichelzusammensetzung (z. B. durch Anticholinergika bzw. Antidepressiva) oder eine Anreicherung von Arzneistoffen im Speichel (Metformin) ursächlich sein. Aber auch die Zellfunktion durch Enzymhemmung (Zytostatika, Statine) oder die Clearance-Funktion abbauender Enzyme (Cytochrom P450 Interaktion) können hier so verändert sein, dass es zur Beeinträchtigung des Schmeckvermögens kommt. Andere mögliche Ursachen sind: Operationen (Tonsillektomie durch eine mögliche Schädigung des Nervus glossopharyngeus, Mittelohr-Operationen durch eine Verletzung oder Durchtrennung der Chorda tympani), Zahnbehandlungen, Infektionen (viral, bakteriell oder Pilz-bedingt), gastro-ösophagealer Reflux, internistische, neurologische oder Vitaminmangelerkrankungen, Erkrankungen der Speicheldrüsen, Chemo- oder Strahlentherapie sowie psychosomatische Ursachen (z. B. larvierte Depressionen).

## Ursachen von Riechstörungen

Die Ursachen von Riechstörungen betreffen eine große Anzahl möglicher Differentialdiagnosen, die sich auf häufige und seltene Krankheiten aufteilen: sinunasal, post-traumatisch, post-infektiös, medikamentös-toxisch, radiogen, neuro-degenerativ, neurologisch, internistisch, iatrogen, und kongenital (Tab. 1). Ein nicht unerheblicher Anteil (10–15 %) bleibt ohne definitive Diagnose (idiopathische Riechstörung), wobei hier auf jeden Fall zum Ausschluss zentraler Ursachen eine MRT-Untersuchung des Schädels durchgeführt werden muss [19].

**Table 1:** 

Ursachen von Riechstörungen	
*Sinunasal*	Chron. Rhinitis/Sinusitis
	Allergie
	Rhinopathia gravidarum
	Septumdeviation
	Tumoren
*Post-traumatisch*	Schädel-Hirn-Trauma, Nasentrauma
*Post-infektiös*	Grippe, COVID-19, common-cold, etc.
*Medikamentös-toxisch*	Z. B. Antibiotika, Psychopharmaka
	Chemotherapeutika
	Gase, Metalle, Industriestäube
	Nasensprays (z. B. Vasokonstriktoren)
*Strahlentherapie*	–
*Neuro-degenerativ*	M. Alzheimer
	M. Parkinson
*Neurologisch*	Insult, Tumoren, z. B: Meningeom
*Internistisch*	Z. B. Leber‑, Nieren‑, Schilddrüsenserkrankungen
*Iatrogen*	Nasen/NNH-OPs, neurochirurgische Ops
*Kongenital*	Isoliert oder syndromal (z. B. Kallmann)
*Idiopathisch*	Ausschlussdiagnose!

Im Rahmen der spezifischen Anamnese werden zunächst die häufigsten Ursachen und der zeitliche Zusammenhang zum Auftreten der Riechstörung abgefragt.

Bei einem ausgedehnten Schädel-Hirn-Trauma (SHT) mit langem Krankenhausaufenthalt stehen anfangs oft andere Beschwerden im Vordergrund und der Patient bemerkt die Einschränkungen des Geruchs- und Geschmackssinns oft erst später, sodass diese auch noch nach Monaten richtig bewusst werden können [20]. Obwohl schwere SHT häufiger zu posttraumatischen Riechstörungen führen, können auch leichte Traumata zu bleibenden Verlusten des Riechvermögens führen. Entgegen der verbreiteten Meinung, hat eine große Studie gezeigt, dass die Anosmie unmittelbar nach einem SHT keine schlechtere Prognose per se besitzt [21]. Als einzige valide Prognosefaktoren haben sich die Dauer und der Schweregrad der Riechstörung sowie das Alter des Patienten gezeigt [22]. Dies bedeutet, dass die beste Prognose (unabhängig von der Art der Riechstörung) der junge Patient mit leichtem, seit kurzem bestehenden Verlust des Riechvermögens und die schlechteste Prognose der ältere Patient mit Anosmie seit langer Zeit hat.

Bei postinfektiösen Riechstörungen (diese sind meist postviral) zeigt sich das Auftreten der Beschwerden unmittelbar nach einem Infekt. Dieser muss nicht unbedingt die Nase betreffen, da systemische Infektionen wie z. B. Influenza oder COVID-19 zu einem Riechverlust auch ohne Einschränkung der Nasenatmung führen können. Obwohl die Patienten oft über schwere oder langwierige Infektionen berichten, schließt ein leichter Infekt keine Riechstörung aus.

## Diagnose von Riechstörungen

Die klinische Untersuchung bei Patienten mit Riech- und Schmeckstörungen beinhaltet den kompletten HNO-Status inkl. der Nasenendoskopie mit Darstellung des mittleren Nasenganges sowie der Riechspalte, soweit diese zwischen Nasenscheidewand und mittlerer Nasenmuschel einsehbar ist.

Die Durchführung einer Bildgebung ist nur dann erforderlich, solange die Diagnose noch unklar bzw. unbedingt notwendig erscheint. So kann bei Verdacht auf sinugene Riechstörung ein diagnostischer Kortison-Versuch über wenige Tage (z. B. 3–5 Tage 1 mg/kgKG Prednisolon-Äquivalent) empfohlen werden. Auf mögliche Kontraindikationen (z. B. akute Magenulzera, schwere Osteoporose und Diabetes mellitus, Depressionen, Glaukom, Myopathien) ist zu achten. Eine Computertomographie der Nasennebenhöhlen sollte bei bloßem Verdacht einer sinugenen Ursache primär nur dann durchgeführt werden, falls kein Kortison verabreicht werden kann bzw. der Patient einer eventuellen Operation der Nasennebenhöhlen zustimmen würde [23]. Demgegenüber hat die Durchführung einer MRT-Untersuchung, neben der Darstellung der zentralen Hirnanteile (v. a. des Stirnhirn inkl. Bulbus olfaktorius), den Vorteil einer zusätzlichen Information über die Nasennebenhöhlen und der Rhinobasis. Damit wird für die Patienten die Strahlenbelastung vermieden.

Ein zentraler Bestandteil der Diagnose von Riechstörungen besteht in der psychophysischen Testung des Riechvermögens mittels validierter Testverfahren. Nur so können die Beschwerden des Patienten objektiviert werden und eine qualifizierte Aussage zum Schweregrad und zur Prognose gemacht werden. Die Verwendung validierter Testmethoden garantiert die Verlässlichkeit und Reproduzierbarkeit der erhaltenen Messwerte. Die in Deutschland, Österreich und der Schweiz sowie bereits in vielen weiteren Ländern verbreitetsten Riechtests sind die Sniffin’ Sticks (Riechstifte, www.burghart.net) [24]. Diese Testbatterie ermöglicht die Messung der Riechschwelle, Geruchsdiskrimination und Identifikation von Gerüchen, mit genauer Bestimmung der Riechleistung im Vergleich mit publizierten Normdaten von über 9000 Probanden [25]. Somit kann eine Einteilung in den Bereich der Normosmie, Hyposmie und Anosmie für den einzelnen Patienten erfolgen. Der Identifikations-Untertest alleine dient als Screening-Verfahren mit kürzerer Testdauer. Außerdem wurden Varianten zur Selbsttestung der Patienten, zur Testung des Geruchsgedächtnisses und zur Wahrnehmung von Duftstoffgemischen sowie ein Kurztest mit wenigen Riechstiften als Kitteltest-Variante und ein Test zur Objektivierung der Parosmie von unserer Arbeitsgruppe validiert und publiziert [26–30].

In Zusammenschau der Anamnese mit dem Ergebnis der Riechtests kann eine erste Arbeitsdiagnose gestellt werden. Bei unklarer Funktion des retronasalen Riechens kann ein validierter Test (z. B. Candy Smell Test) verwendet werden [4]. Dabei kommen Sorbit-Lutschbonbons zur Anwendung, die mit verschiedenen Aromen versehen sind.

Die oben genannten Riech- und Schmecktests werden als psychophysische Tests bezeichnet, die eine aktive Antwort des Patienten oder Probanden benötigen und daher lediglich objektivierenden Charakter haben. Soll z. B. im Rahmen von Gutachten das Riechvermögen mittels objektiver Olfaktometrie bewertet werden, kommen sog. Ereignis-korrelierte Potenziale (OEP-olfaktorisch evozierte Potenziale, z. B. Phenylethylalkohol/Rosenduft, CSSEP-Chemo-somatosensorisch evozierte Potenziale, z. B. CO_2_-geruchloses, schmerzhaftes Gas) zur Anwendung [31].

Hier wird sowohl der Nervus trigeminus als auch der Nervus olfaktorius seitengetrennt mittels EEG-Ableitungen untersucht. Für diese Spezialuntersuchung, die nur an wenigen Zentren durchgeführt wird (siehe AG Olfaktologie und Gustologie der Deutschen HNO-Gesellschaft, www.olfaktologie.hno.org), existieren entsprechende Empfehlungen für die Durchführung [32]. Zu beachten ist, dass das Ergebnis nur in Zusammenschau mit den psychophysischen Tests aussagekräftig ist, da interindividuell gewisse Variationen bestehen.

## Therapie von Riechstörungen

Die Therapie von Riechstörungen richtet sich nach der Diagnose, wobei in erster Linie zwei pathophysiologischen Mechanismen unterschieden werden müssen. Entweder besteht eine beeinträchtigte Duftstoff-Zuleitung zur Riechspalte (*konduktive Riechstörung*) oder es liegt eine Nervenschädigung vor (von den Riechnervenzellen bis zu den relevanten Arealen im Gehirn, *sensorineurale Riechstörung*). Oft ist an eine *Kombination* zu denken (z. B. Schleimhautschwellung und Beeinträchtigung der sensorischen Riechnervenfunktion durch Entzündungsprozesse, z. B. bei allergischer Rhinitis oder chron. Sinusitis).

Somit fällt der HNO-Heilkunde die zentrale Bedeutung in der Behandlung von Patienten mit Riechstörungen zu, da in ihr Fachgebiet die große Gruppe der sinunasalen Erkrankungen mit Beeinträchtigung des Riechvermögens fallen. Insbesondere bei den chronisch entzündlichen Erkrankungen der Nasenschleimhaut (chron. Sinusitis mit/ohne Polyposis nasi) stehen derzeit Zulassungen von Erfolgs-versprechenden Medikamenten bevor (monoklonale Antikörper, z. B. gegen IL4, 5, 13, IgE, z. B. Benralizumab, Dupilumab, Omalizumab). Diese sollen in erster Linie denjenigen Patienten zur Verfügung stehen, die trotz operativer Therapie und/oder systemischer Kortisontherapie unter Symptomen leiden.

Vor der Durchführung chirurgischer Therapiemaßnahmen (z. B. Nasennebenhöhlen-Chirurgie) sollte immer eine systemische Kortisontherapie erfolgen, da die ausbleibende Besserung des Riechvermögens als prognostisch ungünstiges Zeichen einer Verbesserung des Riechvermögens nach einer Operation gewertet werden muss.

Auf jeden Fall müssen Patienten vor jeder chirurgischen rhinologischen Intervention (z. B. Septumplastik, Septorhinoplastik, Nasennebenhöhlen-Chirurgie) darüber aufgeklärt werden, dass sich das Riechvermögen einerseits zwar verbessern, in einem geringeren Prozentsatz aber auch verschlechtern kann, da schon geringste Veränderungen der Strömungsverhältnisse innerhalb der Nase deutliche Einflüsse auf die tatsächliche Duftstoffkonzentration im Bereich der Riechspalte haben können.

Die topische Therapie der Riechschleimhaut kann als nebenwirkungsarme Behandlungsmöglichkeit betrachtet werden, falls Medikamente, wie z. B. Kortison, einen direkten entzündungshemmenden Effekt ausüben sollen. Es hat sich in Studien gezeigt, dass Nasensprays nicht optimal dazu geeignet sind, da diese zu einer Benetzung weit vor der Riechspalte vor allem im Bereich der unteren Nasenmuschel und des Septums führen. Als einfach auszuführende, effektive Variante der Riechspaltenapplikation hat sich die Kaiteki-Position bewährt (japan., angenehm, im Gegensatz zur schwierigen, früher empfohlenen Mekka-Position) [33]. Dabei wird in Rückenlage der Kopf nach hinten und zur Seite geneigt und in das oben liegende Nasenloch eingetropft. So rinnen die Tropfen entlang der Nasenscheidewand in Richtung Riechspalte.

Bei einer rein sensorineuralen Ursache der Riechstörung (z. B. postinfektiöser Riechverlust ohne Schwellung der Nasenschleimhäute) hat sich in Studien mit hoher Evidenz die Therapie mittels strukturiertem Riechtraining als wirksam erwiesen [34]. Medikamentöse Therapieansätze, wie z. B. Vitaminsubstitution haben bisher keine wissenschaftliche Evidenz, obwohl eine Vielzahl an Studien vorliegen, die jedoch nur experimentellen Charakter haben [35].

Wie wird das Riechtraining in der Praxis durchgeführt? Die Patienten sollten zumindest zweimal täglich für jeweils mindestens zwei Minuten an vier verschiedenen Duftstoffen (z. B. Duftöle aus der Drogerie oder Apotheke) riechen und dabei bewusst an die Geruchsqualitäten denken, um sowohl periphere als auch zentrale Regenerationsprozesse zu unterstützen. Es sollten dieselben Duftstoffe für mindestens drei Monate verwendet werden, das Riechtraining gesamt für mindestens 9 Monate bis zu einem Jahr durchgeführt werden. Die Idee zum Riechtraining geht auf Tierversuche zurück, die gezeigt haben, dass bei Mäusen nach Durchtrennung der Fila olfaktoria diejenigen Tiere, die regelmäßig an Duftstoffen gerochen haben, eine schnellere Regeneration des Riechvermögens gezeigt haben.

Zusammenfassend hat sich in den letzten Jahren die Relevanz des menschlichen Geruchssinns für Gesundheit und Lebensqualität sowie dessen außerordentliche Leistungsfähigkeit verdeutlicht. Neben der strukturierten Diagnostik mittels Anamnese, validierter Riech- und Schmecktests sowie bildgebender Verfahren, konnte die Wirksamkeit eines konsequenten Riechtrainings als Therapieverfahren nachgewiesen werden. Zukünftige wissenschaftliche Erkenntnisse werden die molekularen Mechanismen der Riechzellschädigung und deren therapeutische Anwendbarkeit betreffen. Ebenfalls am Anfang der Forschung stehen die Entwicklungen möglicher „Riechimplantate“ in Analogie zu den höchst erfolgreichen Cochlea-Implantaten [36].
